# Handling missed dose of riluzole by population pharmacokinetic model informed remedial regimen

**DOI:** 10.3389/fphar.2026.1890355

**Published:** 2026-09-30

**Authors:** Changlong Liu, Qing Tang, Lingyan Yu, Zhenwei Yu

**Affiliations:** 1 Department of Pharmacy, Hangzhou Linping Hospital of Traditional Chinese Medicine, Hangzhou, China; 2 Research Center for Clinical Pharmacy, Zhejiang University, Hangzhou, China; 3 Department of Pharmacy, The Second Affiliated Hospital, School of Medicine, Zhejiang University, Hangzhou, China; 4 Department of Pharmacy, Sir Run Run Shaw Hospital, School of Medicine, Zhejiang University, Hangzhou, China

**Keywords:** adherence, population pharmacokinetic, remedial dosing, riluzole, simulation

## Abstract

**Background:**

Patients with amyotrophic lateral sclerosis (ALS) frequently exhibit nonadherence characterized by delayed or missed doses while taking riluzole. This study aimed to provide a population pharmacokinetic (PPK) model informed individualized remedial regimens for patients who experience delayed or missed doses of riluzole.

**Methods:**

A PPK model for riluzole was developed via a nonlinear mixed-effect modeling approach based on 3,024 concentration-time points from 72 subjects. The final model was validated by multiple methods. Six remedial regimens for missed doses were simulated for concentration-time profiles under different scenarios, and the optimal remedial regimen was selected based on the least time of concentration beyond the normal range.

**Results:**

The PPK model of riluzole was best described with a two-compartment model with body weight as a covariate of peripheral distribution volume. The typical values of CL/F, V_c_/F, V_p_/F and Q were 67 L/h, 242 L, 376 L and 33.1 L/h, respectively. The model was showed acceptable performance by goodness-of-fit, visual predictive check, bootstrap analysis and normalized distribution prediction errors. Model-based remedial regimens for different missed scenarios were proposed.

**Conclusion:**

A riluzole PPK model was developed and internally evaluated using multiple diagnostic approaches. Model-based remedial dosing strategies for different missed-dose scenarios were identified based on predefined PK criteria and Monte Carlo simulations. These exploratory strategies provide quantitative insights into riluzole exposure during nonadherent dosing conditions.

## Introduction

Amyotrophic lateral sclerosis (ALS), also known as motor neuron disease, is a neurodegenerative disorder primarily characterized by mitochondrial dysfunction and neuroinflammation (Faller et al., 2025; Tsang et al., 2025).It affects both upper and lower motor neuron systems, ultimately leading to generalized muscle weakness and death from respiratory failure (Feldman et al., 2022). Although the triggers of the disease remain unclear, advancing age has been identified as a significant risk factor for ALS (Feldman et al., 2022; GBD 2017 US Neurological Disorders Collaborators et al., 2021). Given the global increase in elderly populations, discovering valuable and effective diagnostic and therapeutic approaches has become imperative. Riluzole was the first drug globally approved for ALS therapy and remained the established treatment for treating ALS (Tzeplaeff et al., 2023; Corcia et al., 2025). Its primary mechanism is believed to involve inhibiting the release of excitatory amino acids such as glutamate, thereby moderately extending patient survival (Andrews et al., 2020).

Riluzole is rapidly absorbed orally, with peak blood concentrations reaches within 1–1.5 h. Approximately 90% is absorbed, although absorption may be affected by high-fat meals (European Medicines Agency, 2026; van Kan et al., 2005). Riluzole has a large volume of distribution (245 ± 69 L), widely distributed throughout the body and capable of crossing the blood-brain barrier (European Medicines Agency, 2026). Most of the drug exists as protein-bound (approximately 97%). The primary metabolic pathway of riluzole involves oxidation by cytochrome P450 1A2 to form N-hydroxy-riluzole (RPR112512), followed by rapid glucuronidation (Wokke, 1996). Studies have reported significant interindividual variability in riluzole exposure, influenced by multiple factors including CYP1A2 activity, non-standardized dosing, smoking status, liver function, and other patient-related variables (European Medicines Agency, 2026; Groeneveld et al., 2001).Therefore, these pharmacokinetic characteristics make model-informed approaches particularly valuable for characterizing exposure variability and evaluating remedial regimens under various clinical scenarios.

In real-world clinical practice, long-term medication adherence significantly impacts the efficacy of chronic drug therapy and ultimately treatment outcomes (Patel et al., 2025). Studies on long-term riluzole adherence among ALS patients have revealed low compliance rates. Beyond medication-related adverse reactions and patients’ lack of confidence in the drug’s efficacy, functional impairments caused by disease progression, such as dysphagia, also contribute to nonadherence (Goutman et al., 2022; Ludolph et al., 2023). Currently, limited information exists regarding administration instructions and management of delayed or missed dose of riluzole. For the prescribing information of riluzole oral tablets approved by the FDA, it is recommended that “If a dose is missed, take the next tablet as originally planned” (RILUTEK, 2008). Similar statements have been approved by the European Medicines Agency (2026). However, these recommendations are primarily based on practical considerations and do not provide quantitative guidance on the impact of different delay durations on riluzole exposure. Although occasional delays or missed doses during chronic oral treatment are common problems, whether a single deviation from the prescribed dosing regimen would lead to clinically significant changes in the exposure level of riluzole has not been systematically studied yet. Recently, population pharmacokinetic (PPK) modeling and simulation approach, which can simulate the concentration profile under different scenarios, are applied to evaluate remedial dosing regimens for various drugs. This study aimed to develop a PPK model for riluzole and evaluate model-informed remedial dosing strategies under scenarios of delayed or missed doses.

## Methods

### Data collection and PPK model development for riluzole

The data used in this study were derived from a previously published crossover pharmacokinetic study aimed at comparing the bioequivalence of two riluzole tablets (Yu F. et al., 2025). The study included 72 healthy adult subjects who received a single dose of each riluzole formulation with a wash period. The subjects’ age ranged from 18 to 50 years (median age of 27 years), and females accounted for 9.7%. The demographic characteristics of the included subjects are shown in Supplementary Table S1. Finally, the final dataset included a total of 72 subjects, with each subject having 42 concentration measurements. Thus, there were 3,024 plasma concentration-time observations available for population pharmacokinetic analysis. No missing concentration data were found. For concentrations below the lower limit of quantification (LLOQ, 0.700 ng/mL), the values were recorded as 0.35 ng/mL (LLOQ/2) and included in the analysis dataset. A nonlinear mixed-effect modeling approach and the software NONMEM (version 7.6.0, ICON Development Solutions, Ellicott City, MD, United States) coupled with PsN (version 5.6.0) and Pirana (version 25.7.1, Certara, Radnor, PA, United States) were adopted to develop the PPK modeling of riluzole. The first-order conditional estimation method with ε-η interaction (FOCE-I) was used for the parameter estimation. R software (version 4.5.2, R Foundation for Statistical Computing, Vienna, Austria) was used to process the NONMEM outputs and visualizations. One- or two-compartment models with first-order absorption and elimination were used to describe the pharmacokinetics of riluzole. The basic model was selected based on objective function values (OFV), Akaike information criterion (AIC) and goodness-of-fit (GOF) plots. The details are shown in Supplementary Table S3.

Sex, age, and body weight were investigated as potential variables affecting riluzole PK. The effects of continuous covariates were modeled using a linear, exponential, or power model, while categorical covariates were modeled using a power model. Body weight was normalized to 75 kg in the final model. The decrease in the OFV of at least 3.84 (p < 0.05) was considered statistically significant for forward inclusion, whereas an increase in OFV greater than 10.83 (p < 0.001) was required for covariate retention during backward elimination.

### PPK model validation

The final PPK model was validated by goodness-of-fit (GOF) plots, bootstrap analysis, visual prediction checks (VPCs) and normalized predication distribution error (NPDE). Scatterplots of observation values vs. individual or population predicted values and conditional weighted residual errors vs. time or population predicted values are included in the GOF plots (Yu B. et al., 2025). For bootstrap analysis, the resampling process was repeated 1,000 times to generated datasets. The medians and 95% confidence intervals of the parameter estimates obtained from repeated bootstrap datasets were compared with the estimates from the final model to assess the robustness and reliability in parameter estimation of the final model (Muthukrishnan et al., 2025). For VPC, 1,000 simulated replicates of the original dataset were performed to evaluate the predictive performance of the final model. The median, fifth, and 95th percentiles of the simulated concentrations were calculated, and the 95% prediction interval for each bin was also derived (Lan et al., 2022). Furthermore, the distribution of NPDE was assessed graphically by plotting NPDE versus time and population predictions, as well as by examining the NPDE density distribution and quantile–quantile (Q–Q) plots.

### Optimal remedial scheme evaluation

During ALS treatment, drug concentrations fluctuate within the model-derived reference concentration range for fully adherent patients. However, if patients delay or miss doses, concentrations deviate from the effective model-derived reference concentration range. Therefore, remedial dosing aims to maintain drug concentrations within the model-derived reference concentration range and minimize the duration spent outside this range. The ALS Therapeutic Drug Monitoring Guidelines do not specify a fixed reference range for the clinical efficacy of riluzole but recommend the use of individual therapeutic ranges. In this study, it is assumed that fully adherent patients achieve the desired efficacy and safety. The reference concentration range was operationally defined as the interval between the 5th percentile of the simulated trough concentration and the 95th percentile of simulated concentrations (1 h) at steady state under a given dosing regimen as in previous studies which describes the middle 90% of the simulated population exposure distribution, rather than representing established efficacy or toxicity thresholds (Li et al., 2023). The choice of 1-h concentration as an indicator is based on the fact that riluzole typically reaches peak plasma concentration approximately 1–1.5 h after oral administration. This is not intended to be equivalent to individual C_max_, but rather serves as a reproducible, time-specific measure of peak exposure, used to define the reference concentration range derived from the model.

Six remedial regimens were proposed, and the concentration–time profiles were simulated 1,000 times (1,000 virtual individuals) for each scenario. The simulations incorporated interindividual variability for CL and V_c_, as well as the body weight effect on V_p_. Interindividual variability for Ka was not included for simulation. Residual unexplained variability was not included because the simulations were designed to evaluate expected PK exposure profiles. Additional simulations were performed for the scenario of a 12-h delayed dose to compare the effects of three remedial doses: 50, 75, and 100 mg. As the source pharmacokinetic study was conducted under fasting conditions, all simulations assumed fasting administration conditions. To evaluate treatment regimens, the total deviation time is calculated as the sum of the deviation time when drug concentrations exceed and fall below the boundary following missed or delayed doses. A delayed dose is defined as administration of the scheduled dose after the planned dosing time but before the next scheduled dosing occasion. Correspondingly a dose that was not administered before the next scheduled dosing time is a missed dose. The regimen with the shortest total deviation time is deemed optimal. Details of six remedial dosing regimens (Regimen A–F) were shown as follows.

- Regimen A: Omit the delayed regular dose (50 mg) and resume the regular dosing regimen (50 mg) at the next scheduled dosing time.
- Regimen B: Administer the delayed regular dose immediately and continue the regular dosing regimen at the next scheduled dosing time.
- Regimen C: Administer a remedial dose (half of the regular dose, 25 mg) immediately, then resume the regular dosing regimen at the next scheduled dosing time.
- Regimen D: Administer the delayed regular dose immediately, followed by a remedial dose (half of the regular dose, 25 mg) at the next scheduled dosing time, then resume the regular dosing regimen.
- Regimen E: Administer both the delayed regular dose and the remedial dose (half of the regular dose, 25 mg) immediately, omit the next scheduled dose, then resume the regular dosing regimen.
- Regimen F: Delay both the delayed regular dose and the remedial dose (half of the regular dose, 25 mg) until the next scheduled dosing time, administer them together, then resume the regular dosing regimen.

## Results

### PPK model development for riluzole

The basic PPK model of riluzole were better described by a two-compartment model than one-compartment based on OFV, AIC and GOF (Supplementary Figure S1). The model included fixed effects of the absorption lag time (ALAG), absorption rate constant (Ka), apparent clearance (CL/F), central compartment distribution volume (V_c_/F), intercompartmental clearance (Q/F), and peripheral compartment distribution volume (V_p_/F). Model development was performed using a stepwise approach. After selecting the structural model, inter-individual variability (IIV) for CL, V_c_, Ka, Q, and V_p_ was evaluated based on OFV, parameter precision, success rate of minimization, and model stability using an exponential inter-individual random effects model. IIV terms associated with unstable boundary or model estimation were excluded, even if only a slight decrease in OFV was observed. Based on these criteria, IIV for CL, V_c_, and Ka was retained, while IIV for Q and V_p_ was removed. Subsequently, ALAG was assessed within a first-order absorption model. The inclusion of ALAG resulted in a significant reduction in OFV (*Δ*OFV = −1,014.799), supporting its incorporation into the final structural model.

Covariate analysis employed a forward inclusion and backward elimination approach to identify potential influencing factors, and the key steps are shown in Supplementary Table S2. Finally, body weight was the only significant covariate in the final model resulting in an improved model fit (*Δ*OFV = -34.622), which was incorporated into V_p_/F as a power function described via Equation 1.

$$V_{p}/F\;\left(L\right)=376\times {\left(\text{BW}/75\right)}^{0.993}$$ (1)

where V_p_/F is the apparent peripheral distribution volume, BW is body weight (kg).

The detailed parameter estimates of the final model are shown in Table 1.

**TABLE 1:** Final population pk parameter estimates and bootstrap results.

Parameter	Unit	Final model results		​	Bootstrap relative bias (%)	1,000 bootstraps Median (95%CI)
		Estimate	Relative SE%	Shrinkage	​	
Fixed effects						
CL/F	L/h	67	4.9%	​	0.13%	67.09 (60.72–73.99)
V_c_/F	L	242	6.3%	​	−0.3%	241.27 (215.42–270.97)
Ka	h^-1^	5.25	19%	​	1.3%	5.32 (3.84–7.68)
Q/F	L/h	33.1	5.4%	​	0.39%	33.23 (29.98–36.76)
V_p_/F	L	376	8.6%	​	0.69%	378.60 (329.59–444.44)
θ_BW_ on V_p_	​	0.993	33.9%	​	2.8%	1.021 (0.561–1.585)
ALAG	h	0.157	1.3%	​	0	0.157 (0.153–0.161)
Random effects						
Interindividual variability (IIV)						
ω CL	​	0.141	15%	1%	−1.4%	0.139 (0.104–0.187)
ω V_c_	​	0.218	16.8%	5%	−1.4%	0.215 (0.152–0.302)
ω Ka	​	0.994	14%	5%	−0.5%	0.989 (0.740–1.291)
Residual error						
Proportional	-	0.127	7%	3%	0	0.127 (0.109–0.144)
Additive	ng/mL	0.0631	30.1%	3%	−1.9%	0.0619 (0.025–0.104)

Abbreviations: Relative SE, relative standard error; 95% CI, 95% confidence interval; IIV, inter-individual variability; CV, coefficient of variation; V_c_/F, apparent central compartment volume; CL/F, apparent clearance; V_p_/F, apparent peripheral compartment volume; Q/F, apparent intercompartmental clearance between the central and peripheral compartments; Ka, absorption rate constant; θ_BW_, estimated power coefficient for the effect of body weight on Vp/F; ALAG, absorption lag time.

### Model validation

The final model was validated by GOF plots, bootstrap analysis, VPC and NPDE. The GOF plots indicate that the model fit is overall acceptable, but some residual variability remains. (Figure 1). The parameter estimates of 1,000 bootstrap runs of the final model, with a 93.9% success rate, are presented in Table 1, supporting the stability of the parameter estimates. In the VPC test, the final model predictions showed no significant bias, and the predicted trends closely matched the observed trends (Figure 2). As part of the model evaluation, the NPDE diagnostic plots are shown in Figure 3, particularly at higher concentrations and during the early post-dose period, with slight deviations observed in the tails of the NPDE distribution.

**FIGURE 1:**
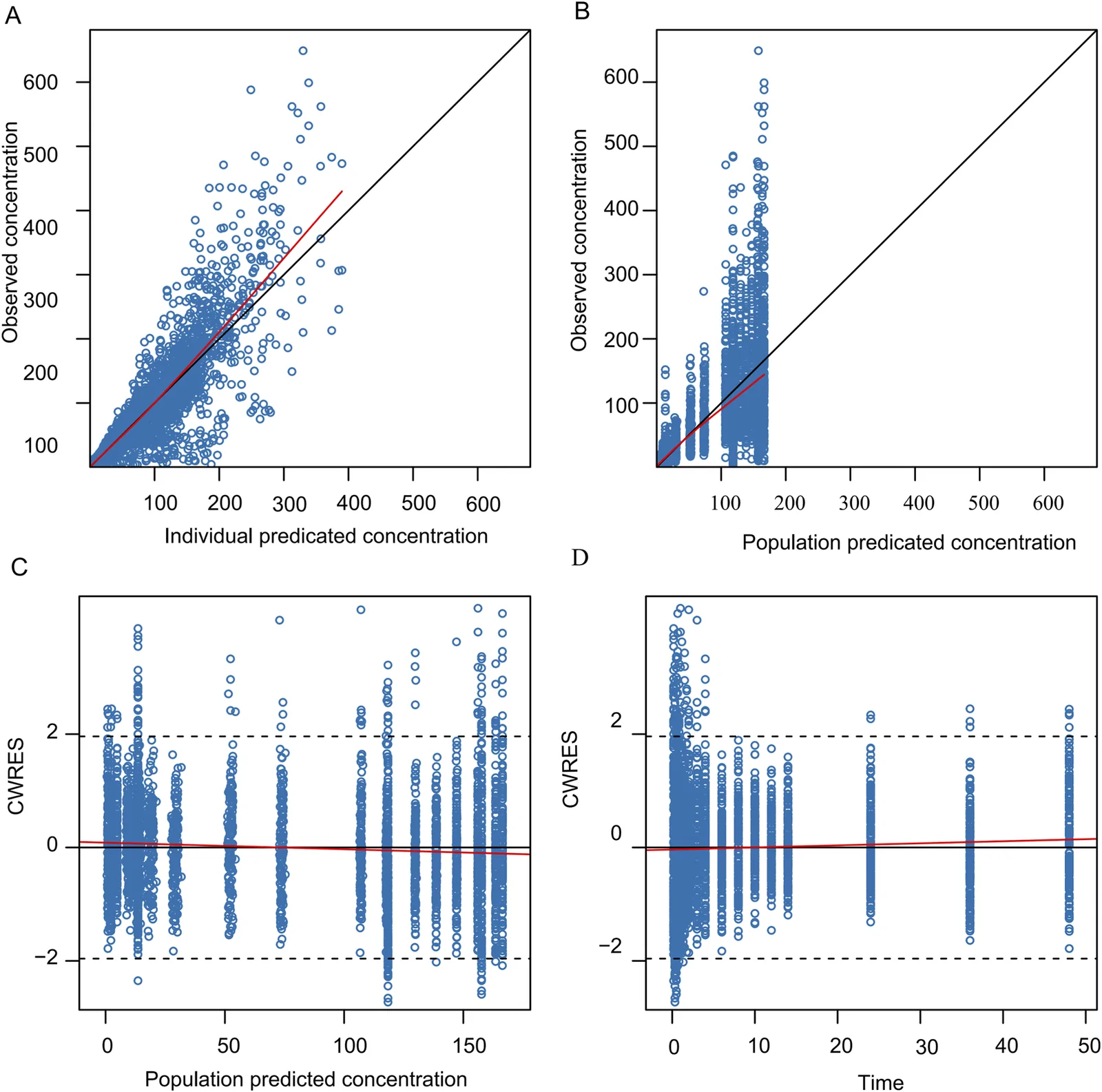
Goodness-of-fit plots for final model of riluzole. **(A)** Observed concentration versus individual predicted concentration; **(B)** observed concentration versus population predicted concentration; **(C)** conditional weighted residuals (CWRES) versus population predicted concentration; **(D)** conditional weighted residuals (CWRES) versus time.

**FIGURE 2:**
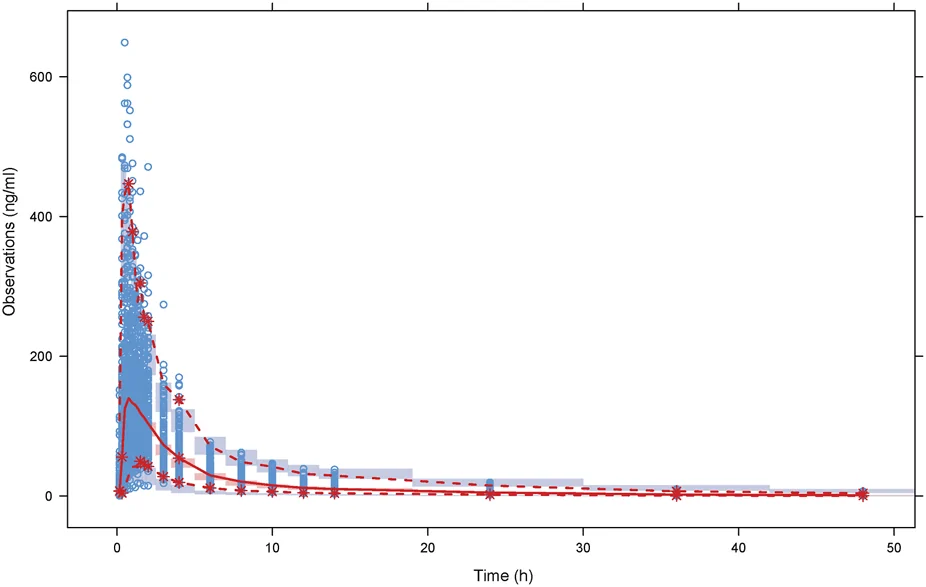
Visual predictive check (VPC)plots of the final population pharmacokinetic model. Open blue circles represent observed drug concentrations. The solid red line represents the 50th percentile (median) of the observed data, and the lower and upper dashed red lines represent the 5th and 95th percentiles of the observed data, respectively. Red asterisks denote the empirical percentiles for each time bin. Observations were binned automatically using the PsN data-density binning algorithm (-auto_bin = auto). Blue shaded areas represent the 95% confidence intervals for the predicted 5th, 50th, and 95th percentiles (from bottom to top) based on 1,000 model-based simulations.

**FIGURE 3:**
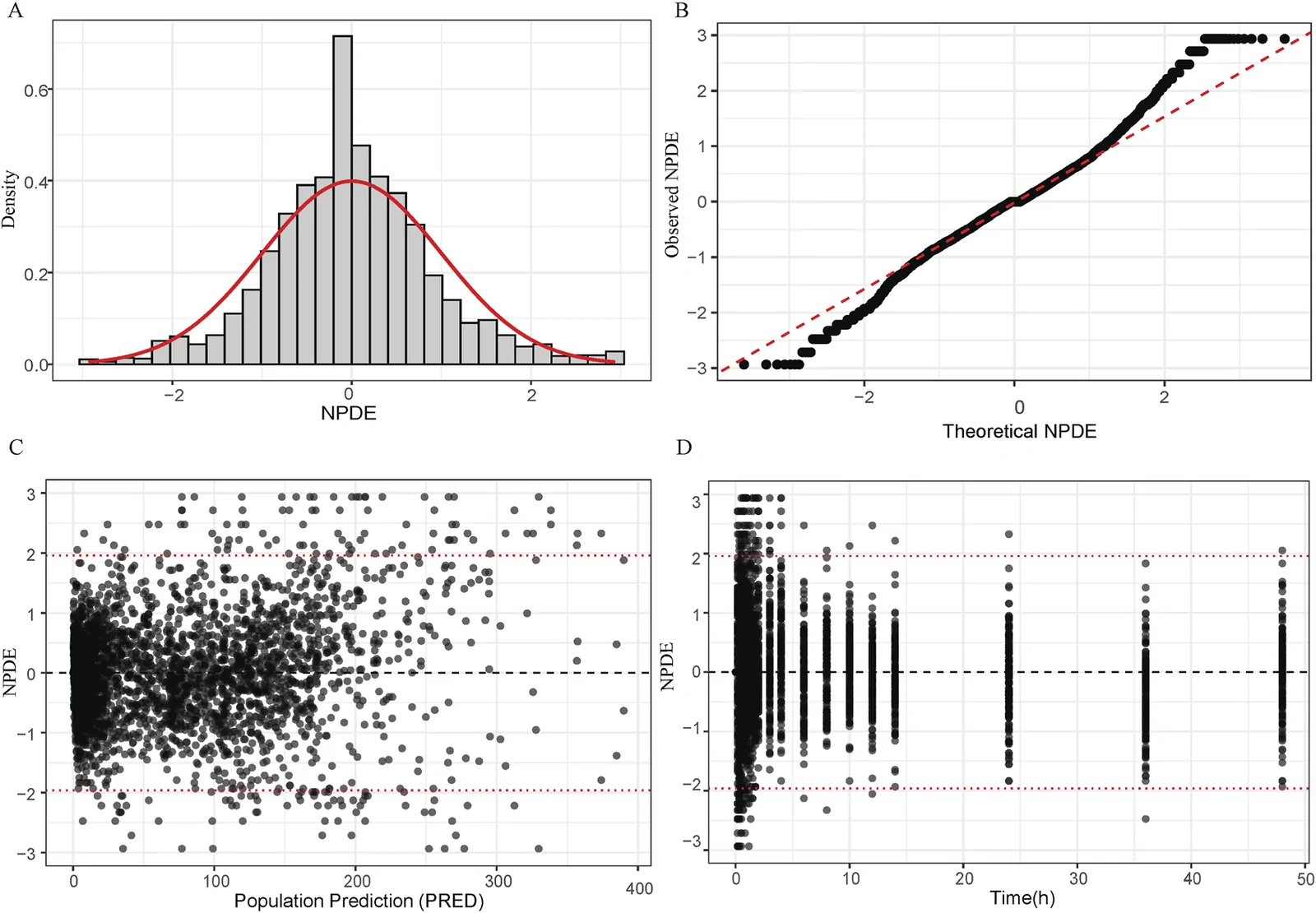
Normalized prediction distribution error (NPDE) check of final model. **(A)** The distribution of NPDE; **(B)** Quantile-quantile plot of NPDE; **(C)** NPDE versus PRED **(D)** NPDE versus Time.

### Remedial dosing recommendations

The steady-state concentration-time curves for candidate remedial regimens are simulated and individualized remedial regimens were evaluated. The curves for the 1-h delayed scenario are shown in Figure 4 as example. And the percentage of time above and below the reference range of each regimen are shown in Supplementary Table S4. We can see that remedial regimen B had the shortest time period of concentration out of the 95% criteria at this delayed scenario (Figure 4B). Taking remedial regimen B as example, the concentration variation at different delay times is shown in Supplementary Figure S2. Based on Monte Carlo simulations of all situations, the optimal remedial regimen results are summarized in Table 2. It can be seen that when the missed dose occurs within 5 h, it is recommended to take the missed dose immediately followed by resumption of the regular dosing regimen at the next scheduled time (Regimen B). When dosing is delayed by 6–10 h, Regimen C is recommended: administer an immediate remedial dose (equivalent to half the regular dose), then resume the regular dose at the next scheduled time. For the 11-h delay scenario, Regimen D was selected based on the prespecified criterion, however, the difference from Regimen C was marginal, with only a 0.4-h reduction in time beyond the reference range. In the scenario of missing the dose for 12 h, the total deviation time for the 75 mg supplementary dose was the shortest (14 h). Although the 100 mg dose was expected to provide a more adequate dose replacement, it resulted in a longer deviation time (27 h), and the simulated C_max_ value was the highest among the doses evaluated. The simulated C_max_ for Regimens E and F reached approximately 500 ng/mL, whereas C_max_ values for the other regimens generally ranged from 300 to 400 ng/mL. Supplementary Table S4 shows the details for Each Regimen simulation results.

**FIGURE 4:**
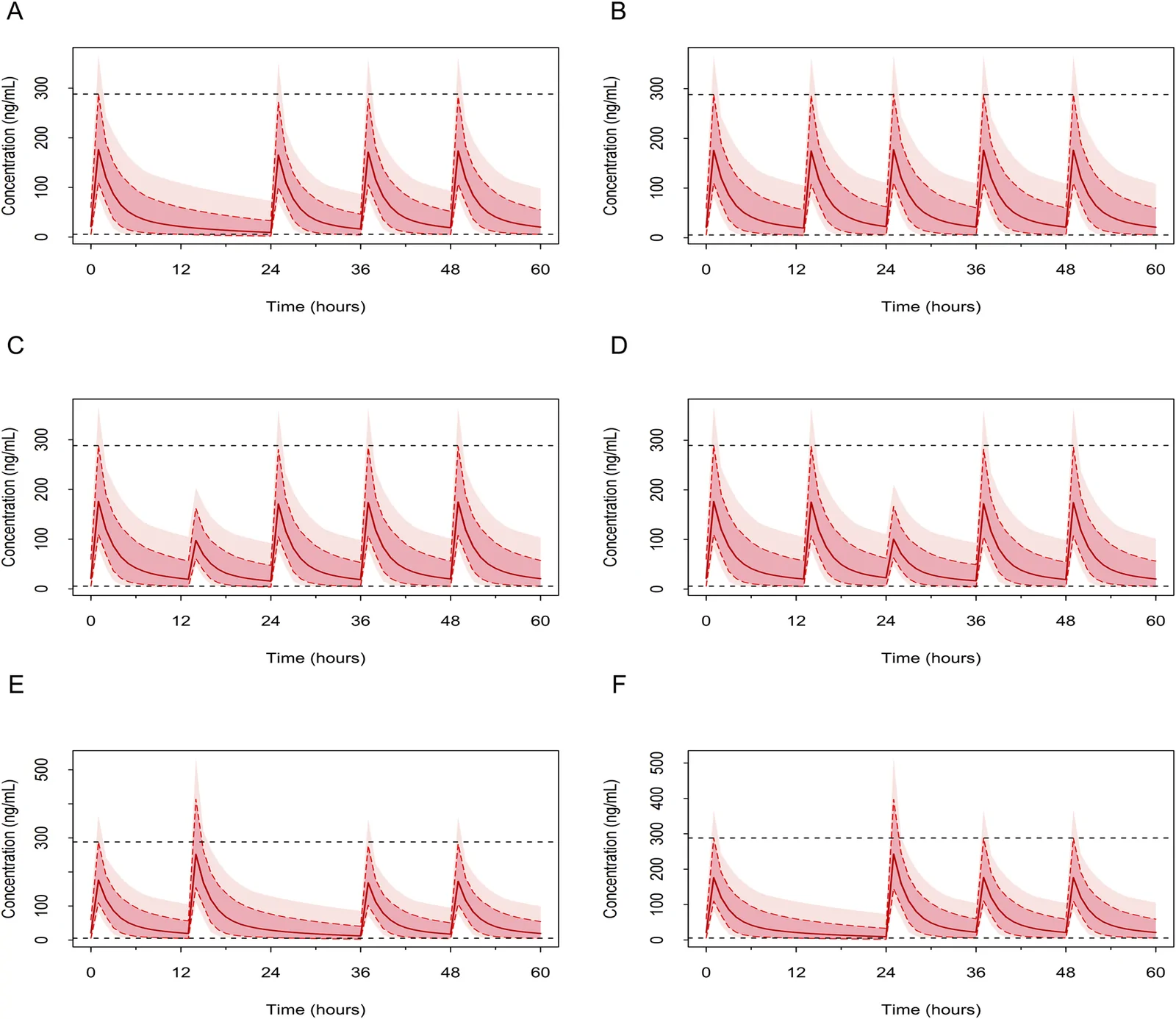
Concentration-time curves of different riluzole remedial dosing regimen for the 1-h delayed scenario **(A)** Concentration-time curve of remedial dosing regimen **(A)** Skip this missed dose and resume the regular dosing schedule at the next scheduled time; **(B)** Concentration-time curves of remedial dosing regimen **(B)** Take the missed dose immediately and resume the regular dosing schedule at the next scheduled time; **(C)** Concentration-time curve of remedial dosing regimen **(C)** Take a remedial dose immediately and resume the regular dosing schedule at the next scheduled time; **(D)** Concentration-time curve of remedial dosing regimen **(D)** Take the missed dose immediately, then take a remedial dose at the next regular time, and resume the regular dosing schedule; **(E)** Concentration-time curve of remedial dosing regimen **(E)** Take the delayed dose and aremedial dose immediately, skip the next scheduled dose, and then resume the regular dosing schedule; **(F)** Concentration-time curve of remedial dosing regimen **(F)** Skip this missed dose and take both a remedial dose and adelayed regular dose at the next scheduled time, then resume the regular dosing schedule; remedial dose = 1/2 scheduled regular dose = 25mg; Assuming the subject takes multiple doses and reaches steady state, the simulation of delayed or missed doses begins at 12 h. The red line represents the simulated median concentration-time curve. The pink shaded area indicates the distribution range from the 5th-95th percentile of simulated concentrations profiles. The light pink shaded area represents the distribution of the simulated concentration profiles.

**TABLE 2:** Recommended remedial dosing regimen under different delayed dosing scenarios.

​	Delayed hours											
^a^Remedial regimen		1 h	2 h	3 h	4 h	5 h	6 h	7 h	8 h	9 h	10 h	11 h
A	Skip **—** 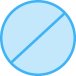	​	​	​	​	​	​	​	​	​	​	​
B	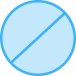 **—** 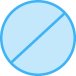	✓	✓	✓	✓	✓	​	​	​	​	​	​
C	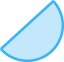 **—** 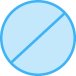	​	​	​	​	​	✓	✓	✓	✓	✓	​
D	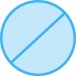 **—** 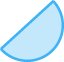	​	​	​	​	​	​	​	​	​	​	**✓**
E	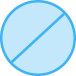 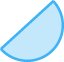 **—** Skip	​	​	​	​	​	​	​	​	​	​	​
F	Skip **—** 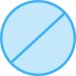 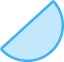	​	​	​	​	​	​	​	​	​	​	​

^a^ Remedial regimen are expressed as “the dose to be taken immediately” — “the dose to be taken at the next scheduled time.“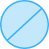, the regular dose; 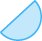, the remedial dose (half of the regular dose); 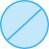 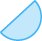, concurrently taking the regular dose and the remedial dose; ✓, the optional regimen.

Given that weight is a covariate of V_p_/F, this study also investigated the influence of body weight on remedial dosing by simulated concentration profiles of virtual subjects with different weights (45kg, 75kg, 100 kg). Typical curves of regimen B at a 2-h and 6-h delay are shown in Supplementary Figure S3, S4. Under fixed delayed dosing scenarios, the selection of the optimal remedial regimen remained consistent across all weight groups.

## Discussion

To the best of our knowledge, this study provides a comprehensive model-informed remedial regimen for missed doses of riluzole for the first time. The PPK model for riluzole was developed, which was validated by multiple methods and was robust. Remedial regimens for missed doses of riluzole were simulated for concentration distribution, and optimal remedial regimens were suggested for different missed dose scenarios.

Typical value of riluzole clearance in this study is comparable to the only available data reported by Bruno et al. (1997). Moreover, the inclusion of body weight as a covariate on the peripheral volume of distribution (V_p_/F) physiologically accounts for the increased tissue capacity for lipophilic drug distribution in heavier individuals (Gouju and Legeay, 2023). Although the PPK model was developed based on healthy subjects’ single-dose data, it could be considered applicable for exposure prediction in ALS patients without major organ dysfunction, while acknowledging potential disease-related differences. Previous studies have shown that riluzole exhibits linear and time-invariant pharmacokinetic properties. Within clinically relevant dose ranges, the study found that both C_max_ and AUC increased proportionally with dose, while elimination half-life remained independent of dose, indicating linear distribution characteristics. Furthermore, repeated-dose studies demonstrated that riluzole reaches steady state within approximately 5 days, with only limited accumulation observed after multiple dosing. (Le Liboux et al., 1997). Another previous exposure-clinical outcome association study had reported the steady state riluzole exposure in ALS patients (Groeneveld et al., 2008). The observed riluzole median peak concentration in steady state was 183 ng/mL, which is highly consistent with our simulation. The trough concentration of 54 (20–343) ng/mL in steady state also falls within an acceptable range compared to our results (Figure 4). In addition, To provide supportive evidence to demonstrate the rationality of the established model in terms of pharmacokinetics, the estimated pharmacokinetic parameters were compared with those reported in previous ALS studies. Bruno et al. (1997)’s model was a one-compartment model, which was high attributed to the sparse sampling strategy. However, the key parameters CLs were highly consistent. This suggests comparable dose-normalized exposure between studies under similar dosing conditions, as AUC is determined by dose and CL/F under linear pharmacokinetics. The half-life of riluzole in these healthy subjects was 11.4 h, and the half-life in ALS patients under multiple doses was also approximately 12 h, which indicated that the effect of tissue/plasma accumulation on exposure was minimal (Yu F. et al., 2025; Bryso et al., 1996).Furthermore, a population pharmacokinetic analysis in ALS patients showed that riluzole clearance was neither dose-dependent (administered twice daily at 25–100 mg) nor related to treatment duration (up to 10 months), confirming the stability of riluzole’s pharmacokinetic properties during long-term therapy (Bruno et al., 1997). Overall, these study results provide strong evidence that the model can reasonably describe riluzole exposure in ALS patients without severe hepatic or renal impairment under clinical dosing conditions, although prospective evaluation in ALS patients receiving repeated dosing is still needed. It is worth noting that the above comparison with ALS research was conducted as an auxiliary cross-study assessment, rather than as a formal external validation.

In addition, Le Liboux et al. (1999) demonstrated that age or gender had no statistically significant effect on riluzole exposure in healthy subjects. Furthermore, although Bruno et al. (1997) reported a lower clearance rate in female ALS patients compared to males, this difference is considered clinically insignificant and does not warrant dose adjustment. Nevertheless, the current study’s dataset has a limited age range and an imbalanced gender distribution, further validation in a more representative ALS population is needed. Additionally, this dataset does not include certain clinically relevant factors that may affect riluzole exposure (such as smoking status, food and liver function), so they cannot be evaluated as covariates. Due to the lack of mealtime information in the model development dataset, the impact of food was not considered in this simulation. Previous study has shown that a high-fat meal reduces the C_max_ of riluzole and delays T_max_, but has only a minor effect on AUC, indicating that food primarily affects absorption kinetics rather than overall exposure . Therefore, variations in meal timing may introduce uncertainty in the predicted peak exposure profiles during dosing.

The optimal remedial dosing regimen in noncompliant situations depends on the half-lives of riluzole in individual patients, and long half-lives reduce the effects of patient nonadherence (Counterman and Lawley, 2021). However, riluzole exhibits a moderate elimination half-life, providing limited buffering against missed or delayed doses, thereby necessitating the development of appropriate remedial dosing strategies to mitigate fluctuations in drug exposure (Yu F. et al., 2025). There is no limited method to prove an optimal remedial regimen as it would be unethical to perform such trials. But it is concerned that skipping dose suggested by the label would increase the risk of low exposure. The Monte Carlo simulation results proved this concern and indicated that the remedial regimen was varied according to the missed dose scenarios. A detailed optimal remedial regimen is proposed by this study, which would benefit the safe and effective use of riluzole. Several studies have suggested remedial dose for other drugs using the same strategy, which indicated that the findings of this study are valuable for clinical practice (Li et al., 2023; Methaneethorn, 2022). Moreover, covariate had no effect on remedial regimen selection, which would simplify the clinical selection. This study demonstrates the application value of quantitative pharmacological models in clinical decision-making. By simulating the changes in drug exposure under different compliance scenarios to design targeted remedial regimen, it further highlights the potential of model-informed strategies in precise drug treatment, and has begun to be gradually applied in other translational medical fields (Zhong et al., 2025; Yao et al., 2026).

But this study also has several limitations. First, the model was developed using data from healthy subjects rather than ALS patients and there is no suitable multiple-dose concentration–time data from patients with ALS for direct external validation. Therefore, the potential effects of concomitant medications on pharmacokinetics were not taken into account. Given that riluzole is primarily metabolized by CYP1A2, drug interactions may alter its pharmacokinetic profile and should be taken into consideration when implementing the remedial dosing recommendations proposed in this study in clinical practice. This limitation implies that the exposure changes and remedial dosing strategies obtained in this study should primarily be regarded as simulation results based on pharmacokinetic models, rather than being interpreted as clinically validated treatment recommendations for ALS patients. Second, this study comprehensively considers the total duration exceeding the individual therapeutic range of the drug, without separately analyzing cases above the upper limit or below the lower limit. Additionally, this study did not address complex scenarios such as multiple consecutive missed doses or long-term adherence fluctuations, whose cumulative effects warrant further investigation. Furthermore, the “individual therapeutic range” as defined in this study should be understood as a baseline operational parameter for simulation. Its primary purpose is to provide a unified evaluation standard for different remedial dosing regimens, it does not represent a clinically validated therapeutic exposure target. Future work will still need to establish exposure–effect relationships in conjunction with clinical outcomes in ALS patients (such as changes in ALSFRS-R scores, survival, and adverse reactions) to validate the clinical applicability of this operational definition and to establish clinically meaningful target exposure ranges.

## Conclusion

This study successfully developed a riluzole PPK model, which was a 2-CMT model with body weight as a covariate of peripheral distribution volume. The model showed adequate performance based on internal evaluation using GOF plots, VPC, NPDE and bootstrapping. Potential remedial regimens were proposed based on predefined PK criteria and Monte Carlo simulations. The proposed strategies may serve as exploratory model-based approaches for managing missed doses, however, their clinical applicability requires further validation in patients with ALS.

## Data Availability

The original contributions presented in the study are included in the article/Supplementary Material, further inquiries can be directed to the corresponding author.
