# Supplementary material for: Handling missed dose of riluzole by population pharmacokinetic model informed remedial regimen
Source: Front Pharmacol. 2026 Sep 30;17:1890355. doi: 10.3389/fphar.2026.1890355 (PMC13661369; doi:10.3389/fphar.2026.1890355)
Supplement: Supplementary file 1 [file DataSheet1.pdf]

Table S1 Characteristics of Studied Subjects

Variable	Mean[a]	Median (Q1-Q3)[a]	Min-Max	No. (%)
Male				65(90.3%)
Age (years)	28.96	27(23-32)	18-50	
BW (kg)	63.87	63.3(58.2-69.5)	45.9-80.1	
Height (cm)	169.29	169.5(163.5-174.5)	146.5-189.5	
BMI (kg/m^2)	22.22	21.8(21-23.6)	19.5-25.7	

Notes: [a]The presented data were baseline data.

Abbreviations: BW: body weight; BMI: body mass index; Q1~Q3: interquartile range

Table S2 Key step in model development procedure

Model No.	Model description	OFV	ΔOFV	p-Value
		Forward inclusion		
1	Base model	19444.15		
2	BW on CL/F	19429.634	-14.516	P<0.05
3	BW on V_p	19409.528	-34.622	P<0.05
4	BW on V_c	19433.835	-10.315	P<0.05
5	SEX on V_p	19433.591	-10.559	P<0.05
6	AGE on CL/F	19443.985	-0.165	P>0.05
7	AGE on V_c	19444.045	-0.105	P>0.05
8	Model 3+ BW on CL/F	19394.711	-14.817	P<0.05
9	Model 3+ BW on V_c	19409.527	-0.001	P>0.05
10	Model 3+ SEX on V_p	19409.219	-0.309	P>0.05
11	Model 3+ AGE on CL/F	19409.369	-0.159	P>0.05
12	Model 3+ AGE on V_c	19409.373	-0.155	P>0.05
13	Model 8+ SEX on V_p	19388.613	-6.098	P<0.05
14	Model 8+ AGE on CL/F	19394.711	0	P>0.05
15	Model 8+AGE on V_c	19394.135	-0.576	P>0.05
		Backward elimination		
16	Removing BW on CL/F from Model 13	19395.539	6.926	P>0.001
17	Removing BW on V_p from Model 13	19423.387	34.774	P<0.001
18	Removing SEX on V_p from Model 13	19394.711	6.098	P>0.001

Abbreviations: V_c: central volume of distribution; V_p: peripheral volume of distribution.; BW: body weight; CL: apparent clearance

Table S3 Base population pharmacokinetic parameter estimate

Parameter	Unit	Base Model Results		
		Estimate	Relative SE%	Shrinkage
Fixed effects				
CL/F	L/h	66.8	4.9%	
V_c	L	244	5.5%	
Ka	h^{-1}	5.1	16.6%	
Q	L/h	32.4	5.1%	
V_p	L	316	5.3%	
ALAG	h	0.157	1.2%	
Random effects				
Interindividual variability (IIV)				
ω^2 CL		0.142	15.1%	1%
ω^2 V_c		0.214	16.7%	5%
ω^2 Ka		0.989	14%	5%
Residual error				
Proportional	%CV	35.5	7.1%	3%
Additive	ng/mL	0.0641	40.2%	3%

Abbreviations: Relative SE: relative standard error; 95%CI: 95% confidence interval; IIV: inter-individual variability; CV: coefficient of variation; V_c: central compartment volume; CL: central compartment clearance; V_p: peripheral compartment volume; Q: inter-compartment clearance between central and peripheral compartment; Ka: absorption rate constant; ALAG, absorption lag time before the drug enters the absorption compartment.

Table S4 Percentage of time beyond the prespecified upper and lower exposure boundaries for each regimen

	Regimen A			Regimen B			Regimen C			Regimen D			Regimen E			Regimen F		
	a	b	t	a	b	t	a	b	t	a	b	t	a	b	t	a	b	t
1h				6.25%	2.08%	8.33%	0%	16.67%	16.67%	0%	14.58%	14.58%	2.08%	27.08%	29.17%			
2h				6.25%	4.17%	10.42%	0%	16.67%	16.67%	0%	16.67%	16.67%	2.08%	25%	27.08%			
3h				6.25%	6.25%	12.50%	0%	16.67%	16.67%	0%	18.75%	18.75%	2.08%	25%	27.08%			
4h				6.25%	8.33%	14.58%	0%	18.75%	18.75%	0%	16.67%	16.67%	2.08%	27.08%	29.17%			
5h				6.25%	10.42%	16.67%	0%	18.75%	18.75%	0%	18.75%	18.75%	2.08%	27.08%	29.17%			
6h				7.50%	10.83%	18.33%	0%	15.00%	15.00%	0%	22.92%	22.92%	2.08%	27.08%	29.17%			
7h				7.50%	12.92%	20.42%	2.08%	15.00%	17.08%	0%	20.83%	20.83%	2.08%	27.08%	29.17%			
8h				7.50%	15.00%	22.50%	2.08%	19.17%	21.25%	1.25%	21.25%	22.50%	2.08%	25.42%	27.42%			
9h				7.50%	17.08%	24.58%	2.08%	17.08%	19.17%	0.208%	23.33%	23.54%	2.08%	25.42%	27.42%			
10h				6.46%	19.17%	25.63%	2.08%	19.17%	21.25%	1.25%	23.33%	24.58%	2.08%	25.42%	27.42%			
11h	0%	37.5%	37.5%	8.33%	21.25%	28.75%	2.08%	21.25%	23.33%	1.25%	21.25%	22.5%	2.08%	25.42%	27.42%	2.08%	25%	27.08%

Abbreviations: a, percentage of time above the reference range, a=hours/48*100%; b, percentage of time below the reference range ,b=hours/48*100%; t, percentage of total time above the reference range, t=hours/48*100%.

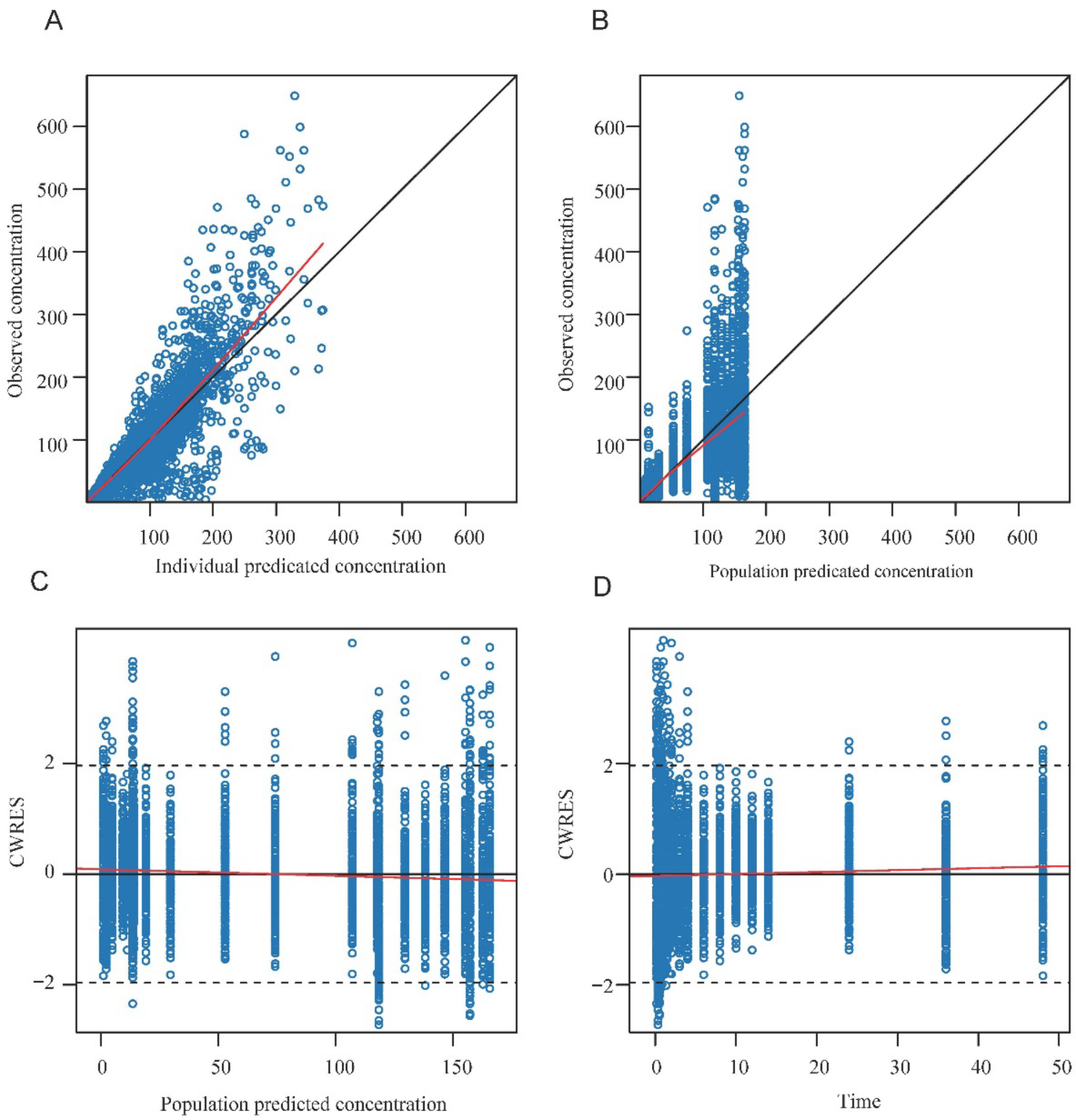

Figure S1 Goodness-of-fit plots for basic model of riluzole.

(A) observed concentration versus individual predicted concentration; (B) observed concentration versus population predicted concentration; (C) conditional weighted residuals (CWRES) versus population predicted concentration; (D) conditional weighted residuals (CWRES) versus time.

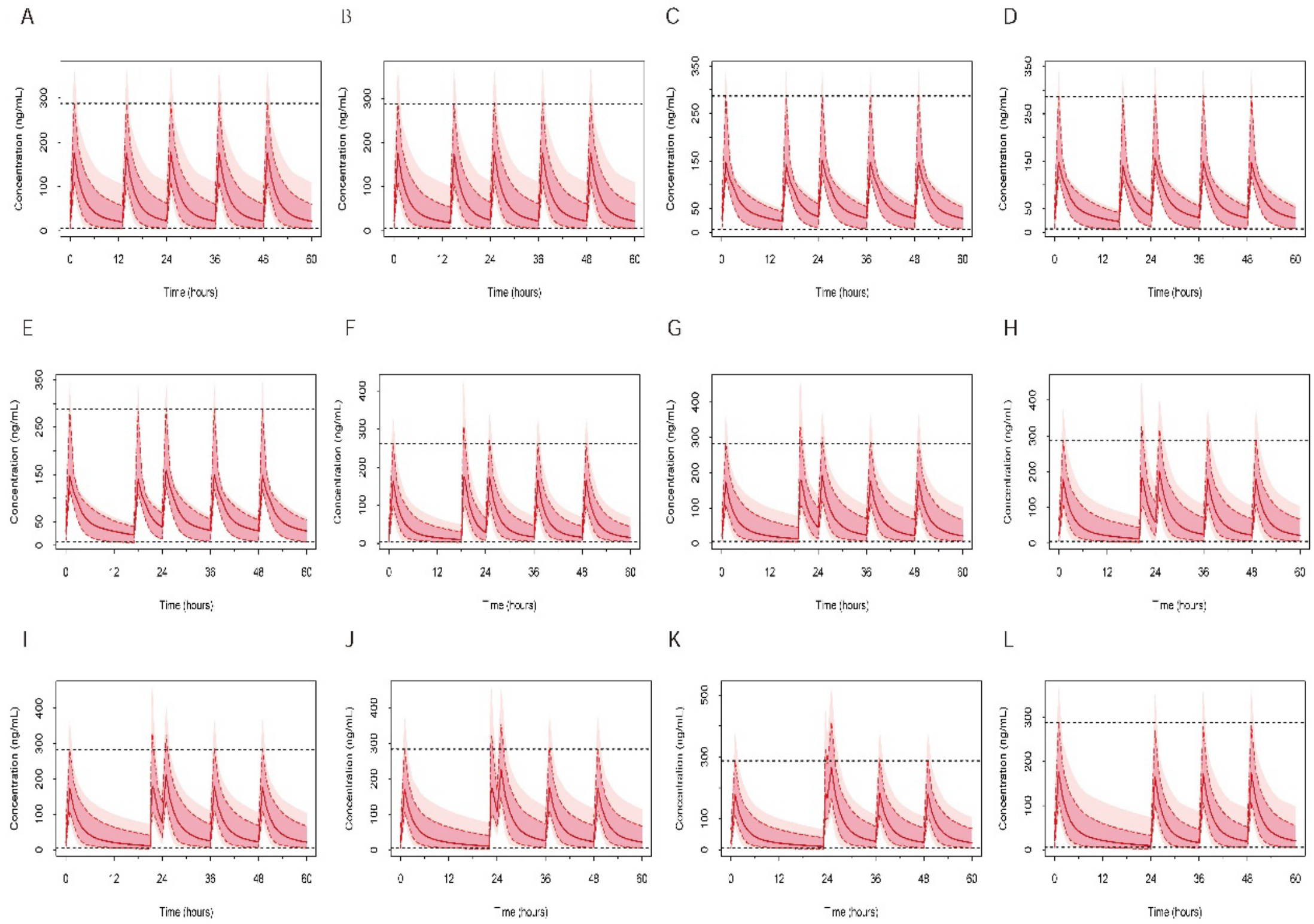

Figure S2 Concentration-time curves of delayed hours from 1 to 12 in regimen B

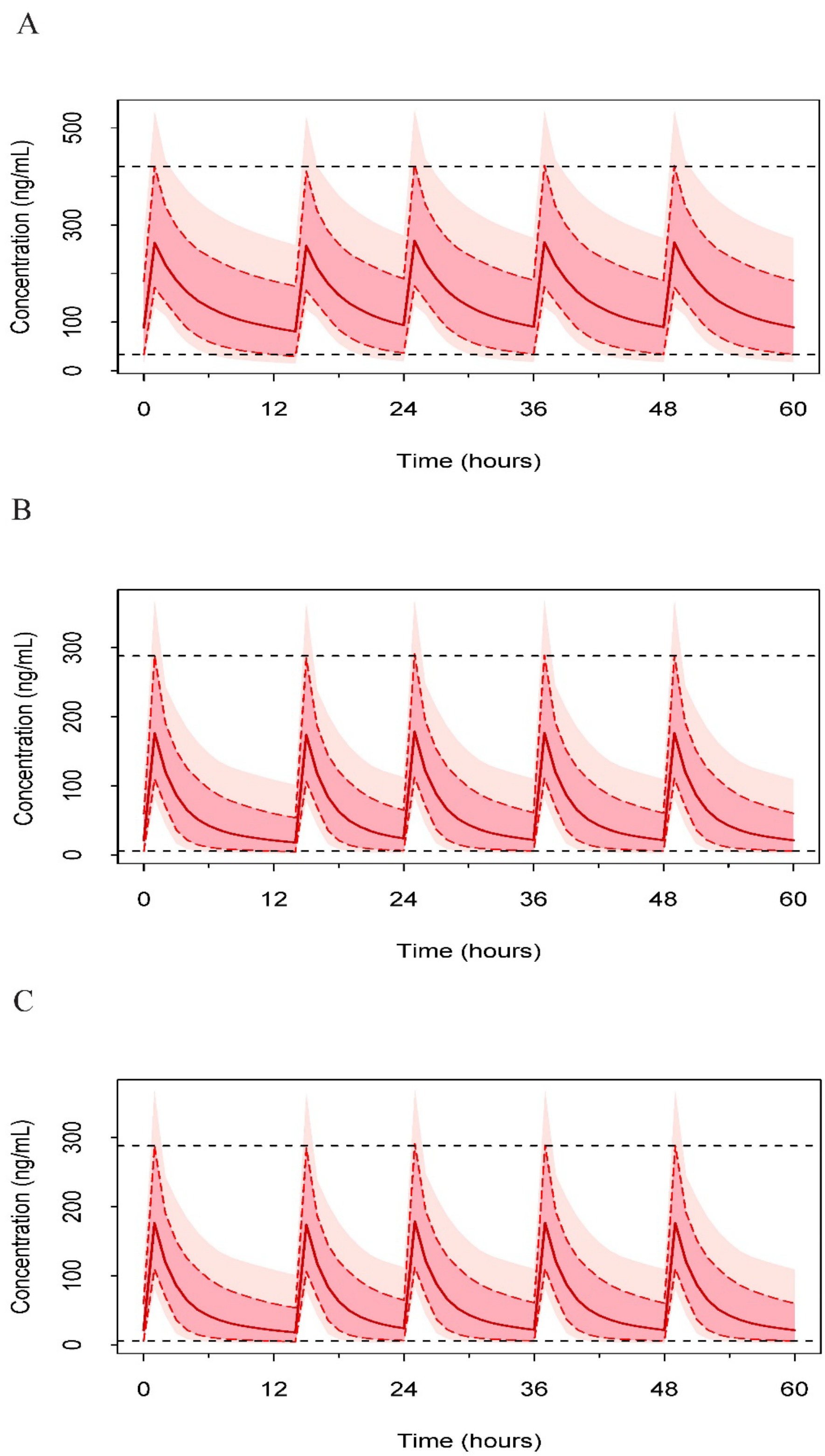

Figure S3 Concentration-time curves of delayed 2 hours in regimen B for subjects weighing 45 kg (A), 75 kg (B) and 100 kg (C).

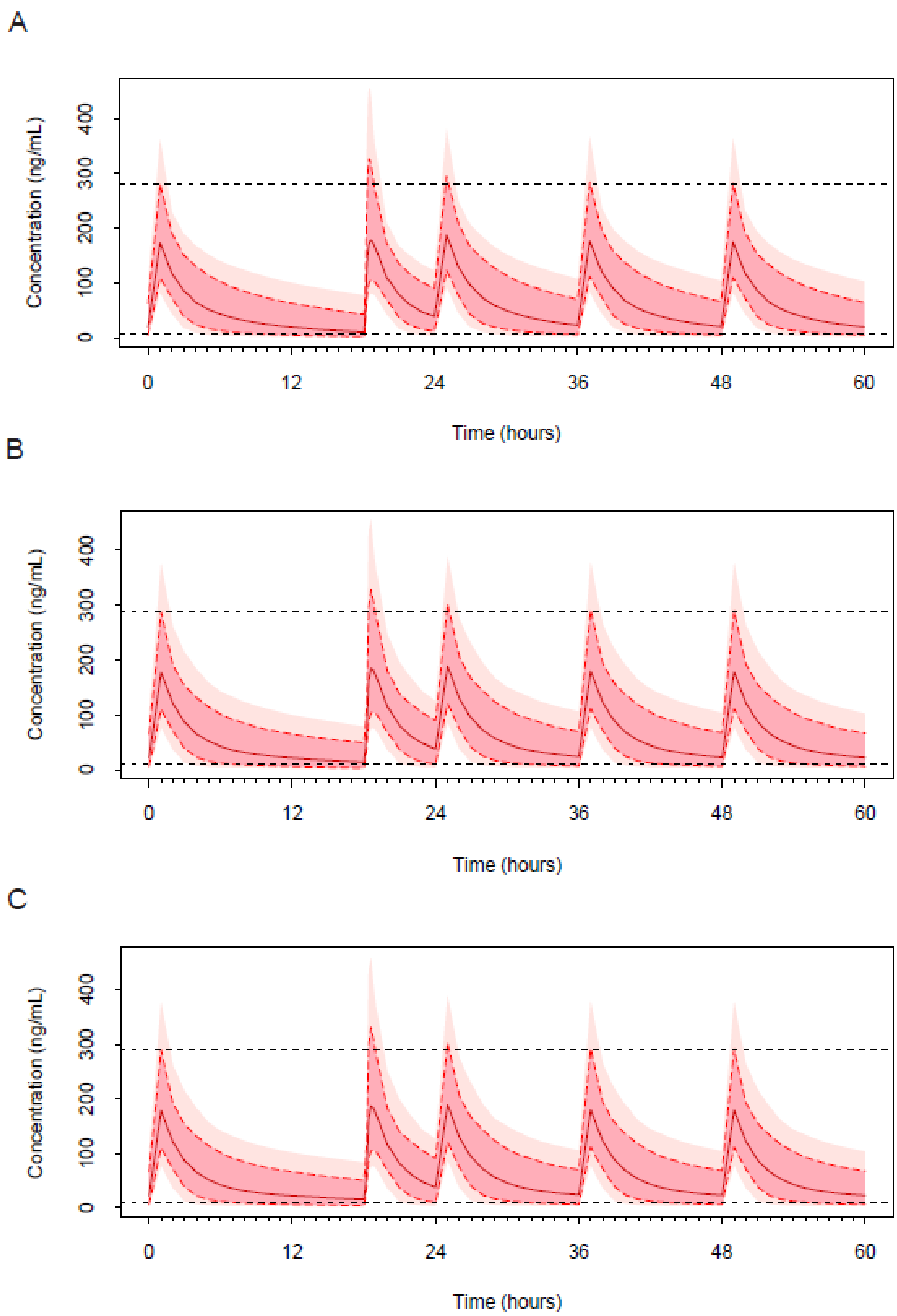

Figure S4 Concentration-time curves of delayed 6 hours in regimen B for subjects weighing 45 kg (A), 75 kg (B) and 100 kg (C)
